# Preliminary Results on Evaluation of Chickpea, *Cicer arietinum*, Genotypes for Resistance to the Pulse Beetle, *Callosobruchus maculatus*

**DOI:** 10.1673/031.009.5801

**Published:** 2009-07-16

**Authors:** F. Erle, F. Ceylan, T. Erdemir, C. Toker

**Affiliations:** ^1^ Department of Plant Protection, Faculty of Agriculture, Akdeniz University, TR-07070 Antalya, Turkey; ^2^ Department of Field Crops, Faculty of Agriculture, Akdeniz University, TR-07070 Antalya, Turkey

**Keywords:** Coleoptera, Bruchidae, seed beetle, genetic resource

## Abstract

The chickpea, *Cicer arietinum* L. (Fabales: Fabaceae), seeds are vulnerable, both in the field and in storage, to attack by seed-beetles. Beetles of the genus *Callosobruchus* are major storage pests of chickpea crops and cause considerable economic losses. In the present study, a total of 11 chickpea genotypes including five ‘*kabuli*’ (Mexican white, Diyar, CA 2969, ILC 8617 and ACC 245) and six ‘*desi*’ chickpeas (ICC 1069, ICC 12422, ICC 14336, ICC 4957, ICC 4969 and ICC 7509) were evaluated for resistance to the pulse beetle *Callosobruchus maculatus* F. (Coleoptera: Bruchidae). Resistance was evaluated by measuring percent damage to seeds. Damage to seeds by *C. maculatus* was manifested by the round exit holes with the ‘flap’ of seed coat made by emerging adults. Of the 11 genotypes tested, only one (ICC 4969) exhibited a complete resistance to *C. maculatus* in both free-choice and no-choice tests; no seed damage was found over the test period. In general, the ‘*desi*’ chickpeas were more resistant to *C. maculatus* than the ‘*kabuli’* chickpeas. Among the tested chickpea genotypes, only ICC 4969 can be used as a source of *C. maculatus* resistance in breeding programmes that could then be grown in organic cultivation free from pesticides.

## Introduction

The chickpea, *Cicer arietinum* L. (Fabales: Fabaceae), is one of the most important grain-legume crops in the world, and the Asia region comprising Turkey contributes 89% of the world chickpea production (Knights et al. 2007). According to the Food and Agriculture Organization (FAO) statistics, cultivated chickpea is in the first rank, with about 10,671,503 ha cultivated, among cool season food legumes in the world and Turkey as well. Like other pulse crops, chickpea is traditionally grown and is an important food and cash crop in Turkey, where it is cultivated on over 557,800 ha annually (FAO 2006). Apart from being an important source of dietary protein for human consumption, this pulse crop is also important for the management of soil fertility due to its nitrogen-fixing ability (Maiti 2001; Kantar et al. 2007).

The chickpea was first culitvated in an area of south-eastern Turkey and adjoining Syria (Toker 2009), but is now cultivated throughout the semi-arid regions of the world (Jodha and Rao 1987; Knights et al. 2007). Cultivated chickpeas are mainly divided into two groups based on plant characteristics and seed size, shape and colouration as ‘*kabui*’ and ‘*desi*’ (Muehlbauer and Singh 1987). The ‘*kabuli*’ chickpeas have relatively large creamy coloured seeds, white flowers and do not contain anthocyanin. In contrast, the ‘*desi*’ chickpeas have small seeds of various colours, purplish flowers and do contain anthocyanin.

The seed-beetles in the genus *Callosobruchus* Pic. (Coleoptera: Bruchidae) are economically important pests of stored pulse crops (van der Maesen 1972; Reed et al. 1987; Weigand 1990; Clement et al. 2004; Demanyk et al. 2007; Sharma et al. 2007). The genus *Callosobruchus* includes approximately 20 species, about three quarters of which are from Asia (Borowiec 1987). These species are cosmopolitan pests of stored legumes (Fabaceae), including the genera *Vigna*, *Phaseolus*, *Glycine*, *Lablab*, *Vicia*, *Pisum*, *Cicer*, *Lens*, *Cajanus* and *Arachis* (Credland 1987; Desroches et al. 1995; Yadav 1997; Ajayi and Laie 2000; Somta et al. 2006). The pulse beetle, *Callosobruchus maculatus* F. (Coleoptera: Bruchidae), is an economically important pest of stored chickpeas, which produces losses up to 30% in a short period of two months (Yadav 1997). Its oviposition and growth are continuous. Females cement eggs to the surface of the host seeds. When eggs hatch, larvae burrow into the seeds where their entire development (four instars plus the pupal stage) is completed. Larvae cannot move among seeds and are thus restricted to the seed on which the female oviposited. Beetles emerge from seeds reproductively mature. Emerging adults are well adapted to storage conditions, requiring neither food nor water to reproduce (Messina 1991). Infestation with the seed bettle was reported to be up to 100% in many stored chickpea (Weigand and Pimpert 1993). When an infestation of 40–60% in chickpea occurs, the seeds are no longer edible (van der Maesen 1972). Because infestation by beetles most commonly occurs in stored seed, laboratory conditions do not significantly differ from their natural conditions (Southgate 1979).

In Turkey, conventional treatments have been used in protection of stored chickpeas against bruchid species, but now other ecologically sound methods based on the use of resistant genotypes are needed for an integrated approach to pest management. Therefore, the present study on evaluation of different chickpea genotypes for resistance to the *C. maculatus* was aimed at finding resistant chickpea genotypes for the management of this pest species through use of resistance in stored chickpeas.

## Materials and Methods

### Test chickpea genotypes

A total of 11 *C. arietinum* genotypes including five ‘*kabui*’ (Mexican white, Diyar, CA 2969, ILC 8617 and ACC 245) and six ‘*desi*’ (ICC 1069, ICC 12422, ICC 14336, ICC 4957, ICC 4969 and ICC 7509) chickpeas were used in the present study. The test chickpea genotypes were supplied by the International Crop Research Institute in Semi Arid Tropics (ICRISAT), the International Center for Agricultural Research Areas (ICARDA) and the Aegean Agricultural Research Institute (AARI), and their characteristics are presented in Table 1. Prior to testing, all test genotypes were kept for two days in an incubator at 26 ± 2° C, 65 ± 5% RH and a photoperiod of 12:12 L:D.

### Test insects and maintenance

Test insects used in the present investigation were obtained from a laboratory culture of *C. maculatus* maintained for 2 years at the Plant Protection Department, Akdeniz University, Antalya, Turkey. Rearing was done on a diet including *C arietinum* seeds at 26 ± 2° C and 65 ± 5% RH in complete darkness.

### Resistance tests

Test chickpea genotypes were screened for resistance to the *C. maculatus* in both free-choice and no-choice tests under laboratory conditions.

### Free-choice test

**Figure 1.**
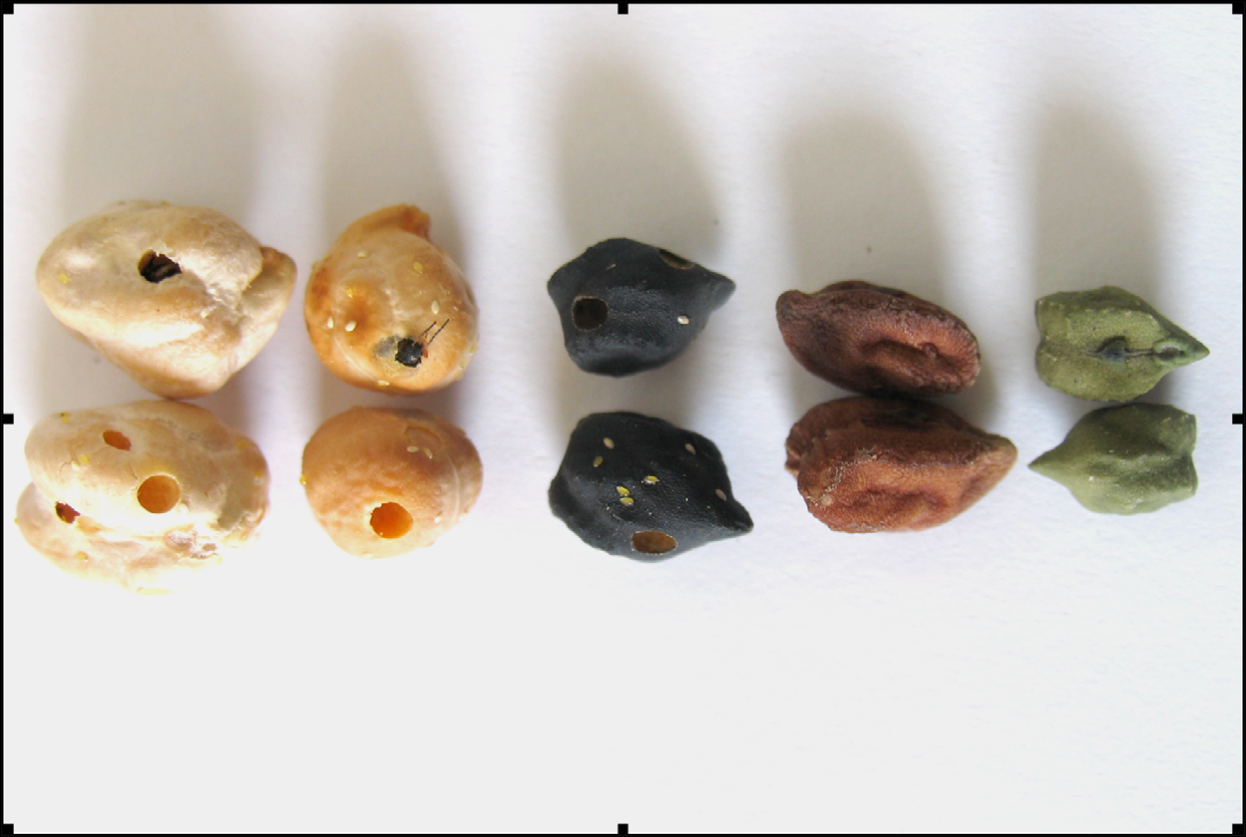
Adult emergence holes and eggs of *Callosobruchus maculatus* on seeds of two ‘*kabuli*’ and three ‘*desi*’ chickpeas (from left to right).

In free-choice test, all test *C. anetinum* genotypes were subjected to the attack of *C. maculatus* freely, following the method described by Raina (1971) and Dahms (1972). For this test, three seeds of each genotype (i.e. 3 × 11 = 33 seeds in total) were placed in each plastic jar of 11 × 9.5 cm size. Each jar was considered as one replication and three replicates using different genotypes were performed for free-choice test. Ten pairs of 0–24-h-old adults of *C. maculatus* were collected from the maintained culture and released in each jar. The jars were covered with muslin cloth, the rim of the lid was placed on the jar so as to avoid the escape of *C. maculatus* adults, and provide air circulation. The insects were allowed to remain there for the purpose of oviposition for one week, and were then removed. The genotypes were examined on biweekly basis to record the number of damaged seeds per genotype by visual observation. Damage to seeds by *C. maculatus* was manifested by the round exit holes with the ‘flap’ of seed coat made by emerging adults (Figure 1) (Ahmed et al. 1989; Riaz et al. 2000). Final observations of grain damage were recorded seventy days after release of *C. maculatus*. The percent grain damage was calculated following Khattak et al. (1987) seventy days after release of *C. maculatus*.

**Table 1.**
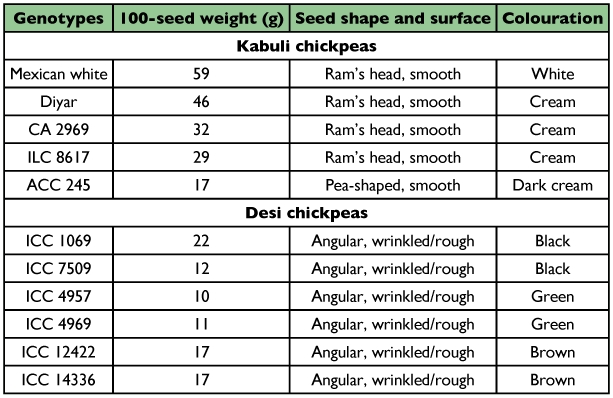
Standard specifications of the test chickpea genotypes used in the study

**Figure 2.**
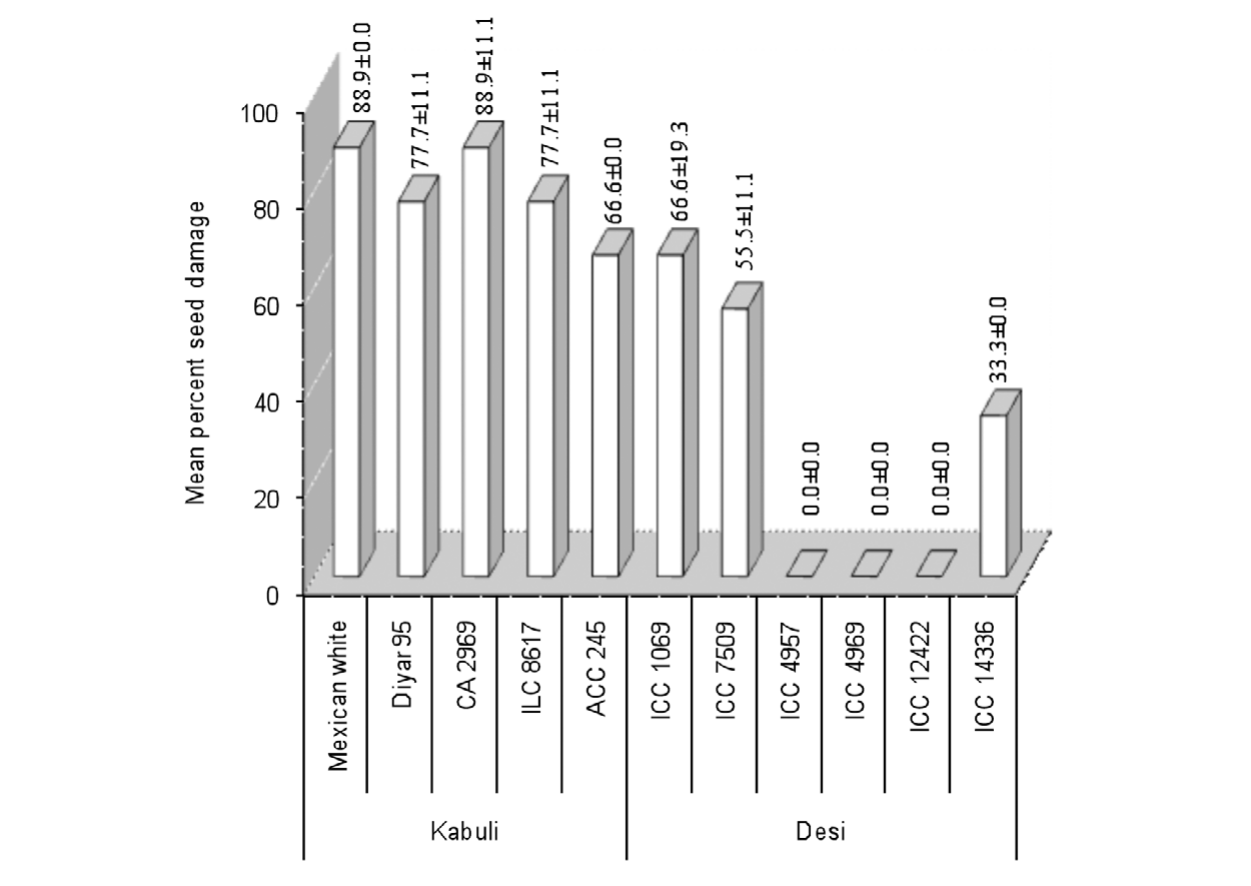
Percentage seed damage rates of different chickpea genotypes, screened for *Callosobruchus maculatus* resistance in free-choice test (bars show means ± standard errors).

### No-choice test

In this test *C. maculatus* were allowed access to only one seed genotype. Thirty-three seeds of a genotype were placed in a jar of 11 × 9.5 cm size, and each jar was considered as one replication for each genotype. This test was carried out using three replications of all 11 chickpea genotypes. Ten pairs of 0–24-h-old adults of *C. maculatus* were released into each jar in each replication. After a one-week allowance for oviposition, the insects were removed, and then the same procedure was followed as in the free-choice test. The genotypes were checked at biweekly intervals to determine the incidence of seed damage by *C. maculatus*. The percent seed damage was calculated after seventy days of the release of *C. maculatus*.

In both free-choice and no-choice tests, seed damage was expressed as the percentage of damaged seeds for each genotype, and the percentage damage incidence was determined using the formula described by Khattak et al. (1987):

% damage incidence=(Number of seeds damaged/ Total number of seeds) × 100

The percentage of seed damage was also calculated according to Weigand and Tahhan (1990) and Singh et al. (1998) with some modifications as follows: 0% = completely resistant or immune (no holes are available), 1–9% = resistant, 10–69% = moderately susceptible, 70–99% = highly susceptible, 100% = completely susceptible.

### Statistical analysis

The data recorded in all the tests were converted to percentages in order to perform analysis of variance using MINITAB.

## Results

### Free-choice test

Statistically significant differences in seed damage were observed among the chickpea genotypes (P≤0.01). Of the eleven genotypes tested, only three ‘*desi*’ chickpeas (ICC 4957, ICC 4969 and ICC 12422) exhibited a complete resistance to *C. maculatus* in free-choice test. Three ‘*desi*’ (ICC 1069, ICC 7509 and ICC 14336) and one ‘*kabuli*’ (ACC 245) chickpeas were found to be moderately susceptible. The remaining genotypes (Mexican white, CA 2969, Diyar 95 and ILC 8617), all of which are ‘*kabuli*’ chickpeas, were categorized as highly susceptible (Figure 2).

**Figure 3.**
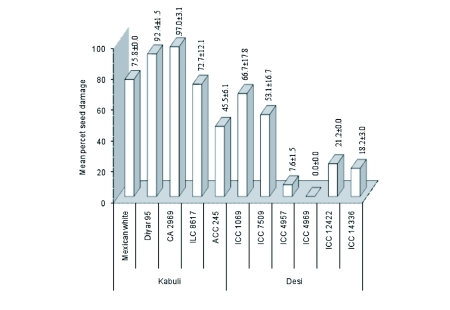
Percentage seed damage rates of different chickpea genotypes, screened for *Callosobruchus maculatus* resistance in no-choice test (bars show means ± standard errors).

### No-choice test

In this test, genotypic effects were found to be statistically significant for seed damage by *C. maculatus* (P≤0.01). Only one genotype from ‘*desi*’ chickpeas, ICC 4969, was observed to be completely resistant to the *C. maculatus.* Another ‘*desi*’ chickpea, ICC 4957, was less resistant. The remaining four genotypes of ‘*desi*’ chickpeas (ICC 14336, ICC 12422, ICC 7509 and ICC 1069) and one genotype from ‘*kabuli*’ chickpeas (ACC 245) were shown to be moderately susceptible to the *C. maculatus* in no-choice test. The rest of the ‘*kabuli*’ chickpeas (CA 2969, Diyar 95, Mexican white and ILC 8617) were recorded as highly susceptible (Figure 3).

Of the eleven chickpea genotypes tested, ICC 4969 was the only chickpea genotype that was found to be completely resistant or ‘immune’ to the *C. maculatus* in both free-choice and no-choice tests as neither seed damage nor holes were observed during the study (Figures 1–2). When ‘*desi*’ and ‘*kabuli*’ chickpeas were compared in terms of seed damage in general, the *desi* genotypes were more resistant to *C. maculatus* than the *kabuli* genotypes (Figures 1–3).

## Discussion

Certain factors such as seed hardness, small seed size, absence of nutritional factors, and presence of toxic substances, may affect bruchid damage to legume seeds (Southgate 1979). Our results implied that especially rough (wrinkled) and thick seed coat might be responsible for resistance to the test bruchid species.

Raina (1971) found that the chickpea strain named G109-1 was significantly better than other varieties in being least preferred for oviposition by seed-beetles. G109-1 had a rough seed coat that is almost spiny (Raina 1971). All accessions of *Cicer echinospermum* P.H. Davis, most of accessions of *C. bijugum* K.H. Rech, and some accessions of *C. reticulatum* Ladiz, were found free from damage (Weigand and Pimbert 1993; Singh et al. 1998) due to their echinate, spiny and tuberculate seed coat, respectively.

In the present study, two genotypes, ICC 4969 and ICC 4957 showed resistance to *C. maculatus*, the former appeared to be completely resistant or ‘immune’ to the test insect species in both free-choice and no-choice tests, and the latter exhibited complete resistance in free-choice test, but had a seed damage of 7.6% in no-choice test. These seeds were smaller in size than the rest of test chickpea genotypes (100-seed weights being 11 and 10 g, respectively). Moreover, these two genotypes were coloured green. Riaz et al. (2000) found that NCS-960003 and Bittle-98 chickpea genotypes were partially resistant to *Callosobruchus chinensis* L.

Reed et al. (1987) reported that many studies have been made to select chickpeas that are resistant to *Callosobruchus* spp., and the ‘*kabuli*’ chickpeas appear to be the most susceptible to *Callosobruchus* spp. More than 3000 ‘*kabuli*’ chickpeas were screened for resistance to *C. chinensis* at the International Center for Agricultural Research Areas, but no resistant germplasm sources were found (Reed et al. 1987). The ‘*desi*’ chickpeas with thick, rough or tuberculate seed coats were found to be resistant but none of them were found to be ‘immune’ or free from damage (Reed et al. 1987). In the present study, the ‘*kabuli*’ chickpeas, in general, were more susceptible to the *C. maculatus* than the ‘*desi*’ chickpeas. However, unlike the findings of Reed et al. (1987), the genotype ICC 4969 proved to be completely resistant or ‘immune’ against the *C. maculatus* in our study. Meena et al. (2004, 2005) studied genetics of seed shape and seed roughness in chickpea and found that ‘*desi*’ chickpeas were dominant over both ‘*kabuli*’ and ‘*pea*’ chickpeas and rough seed surface was dominant over smooth seed surface. The seed characteristics of ICC 4969 could be easily transferred into ‘*kabu*’ chickpeas; however, such “unsightly” seeds may be unacceptable to consumers (Reed et al. 1987; Clement et al. 2004) especially in ‘*kabuli*’ chickpea growing areas in the world. In contrast, it may be acceptable in many areas of the world where ‘*desi*’ chickpeas are mainly grown.

Although control of the pest during storage is possible using methods such as commercial chemicals, irradiation, diatomaceous earth, heating and the grading system (Yadav 1997; Keita et al. 2000; Chauhan and Ghaffar 2002; Demanyk et al. 2007), the most environmental friendly and reliable method is used resistance sources. The results of this study show that the genotype ICC 4969 is a promising one which can be incorporated in future breeding programmes as bruchid-resistant chickpea line, and this genotype also deserves further studies as it is free from damage by the seed beatle.
